# Dementia with aphasia and mirror phenomenon: examination of the mechanism using neuroimaging and neuropsychological findings: a case report

**DOI:** 10.1186/s12883-020-01994-9

**Published:** 2020-11-24

**Authors:** Aiko Osawa, Shinichiro Maeshima, Hidenori Arai, Izumi Kondo

**Affiliations:** 1 Department of Rehabilitation Medicine, National Center for Geriatrics and Gerontology, 7-430 Morioka-cho, Obu, Aichi 474-8511 Japan; 2 Kinjo University, Hakusan, Ishikawa, Japan; 3 National Center for Geriatrics and Gerontology, Obu, Aichi, Japan

**Keywords:** Mirror phenomenon, Aphasia, Dementia, Neurological imaging, Neuropsychological analysis

## Abstract

**Background:**

Aphasia often appears in persons living with dementia; however, aphasia and the mirror phenomenon are rarely present at the same time.

**Case presentation:**

Here, we report a case of fluent conversation with a person in a mirror or a magazine, and examine the underlying mechanism using brain imaging and neuropsychological findings. We found that the appearance of the mirror phenomenon may be associated with a visuospatial dysfunction caused by a decreased function of the posterior region of the right temporal and parietal lobe. Moreover, active talking to a person in a mirror or a person in a magazine could be associated with disinhibition caused by a decline in bilateral frontal lobe function.

**Conclusions:**

This case represents a very valuable and interesting presentation because it is the first report of a long-term follow-up of the course of dementia using neurological imaging, and of the neuropsychological analysis of the mechanism of conversation with a mirror image combined with aphasia.

## Background

The “mirror phenomenon” is the phenomenon of interacting with one’s mirrored self-image by misidentifying one’s own reflection in the mirror as another person, by talking or handing something to the image. Since its first report by Kahn (1925) [1], studies have addressed the mirror phenomenon in Alzheimer’s disease, traumatic brain injury, and vascular dementia and have reported a prevalence of 2.3–5.4% [2–5]. Conversely, aphasia is a core symptom of dementia, and communication problems often appear after the middle stage of this disease. Here, we report a case of Wernicke’s aphasia with acquired stuttering (AS) that exhibited a specific speech pattern after the appearance of the mirror phenomenon during the course of dementia with aphasia. Moreover, based on imaging findings, we also studied the mechanism underlying the mirror phenomenon, as well as the effects of this phenomenon on conversation.

## Case presentation

The present case was a right-handed female with 12 years of education who had been sociable and talkative.

### History of the present illness

At the age of 68, the patient was referred to our hospital because she occasionally burnt her food while cooking. She had no special medical history or family history and was not on medication. Her mini-mental state examination (MMSE) score was 28 (score on the delayed recall domain, 1/3) and she was diagnosed with mild cognitive impairment because of the absence of significant problems in her activities of daily living (ADL). The following year, she exhibited progression of the memory disturbance and was started on donepezil for the management of Alzheimer’s disease. Two years later, she exhibited mild word amnesia and word-finding difficulty, and a decline in auditory comprehension of long sentences. She was referred to our department of Rehabilitation Medicine when she was 72 years old.

### Neurological findings (at the age of 72)

At the first visit, she showed good alertness but was disoriented regarding time and place. Her visual field was normal, and there were no abnormalities in other cranial nervous systems. Moreover, there was no motor paralysis or sensory disturbance. Her deep tendon reflex was normal and no pathological reflexes were observed. She could walk and perform basic movements around herself independently.

### Neuropsychological findings (at the age of 72)

Her spontaneous speech was fluent, but there were word-finding difficulties when addressing others, and phonemic paraphasia was observed. She had word amnesia, and demonstrative pronouns were frequently used, such as “What is that? That, that.” Word repetition was intact, but she could not repeat long sentences, and the percentage of correct answers of sentence repetitions was approximately 80%. Moreover, word comprehension was intact, but sentence comprehension was deteriorated. She sometimes was unable to recall characters of Kanji and Kana on dictation. There was no apraxia. Face recognition of both known and unknown individuals was retained, and she could distinguish facial expressions, age, and gender.

### The course of aphasia and visual cognitive impairment (from the age of 73)

**Fig. 1:**
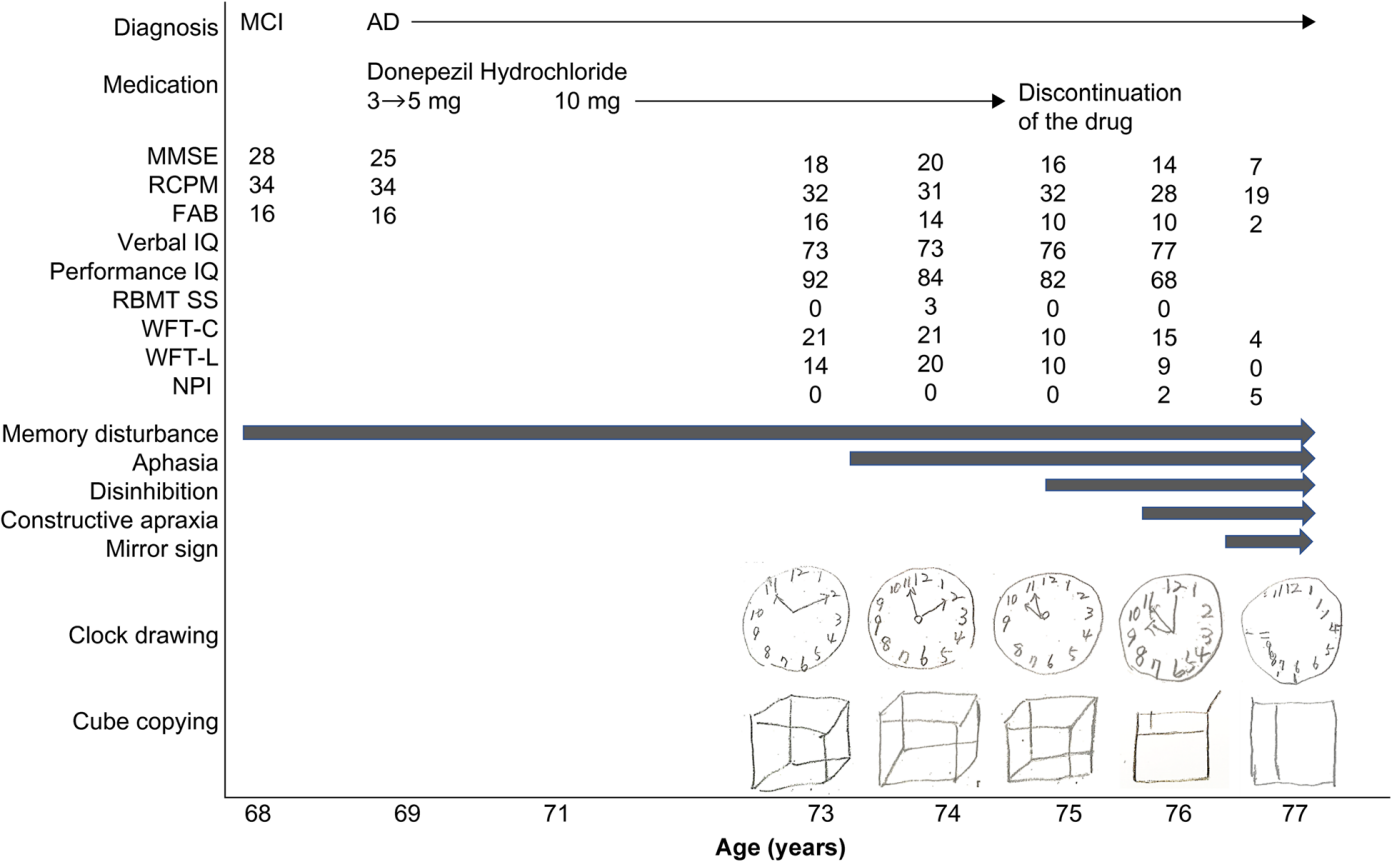
Clinical course of the patient from the onset of the disease. The diagram shows the flow of neurological and neuropsychological processes from the onset of the disease to the appearance of the mirror phenomenon

Figure 1 summarizes the course of the disease from its onset. At the age of 74, in addition to fluent spontaneous speech, the frequent use of demonstrative pronouns, as well as roundabout explanations and phonemic paraphasia, were observed. The percentage of correct sentence repetitions was approximately 50%.

At the age of 75, nausea appeared and donepezil was discontinued. From around this time, her auditory comprehension declined further and she often asked for repetition. In addition, disinhibited attitudes appeared, such as an uncontrollable urge to reach out and to touch things in front of her, the lack of ability to hold herself quiet in a group, and touching the shoulder or back of the attending physician like a close friend.

At the age of 76, she faced difficulties uttering meaningful words in her spontaneous speech, which was a mixture of jargons and stuttering, such as “kyo, kyo, kyo, kyou, kyou, gen, ge, ge, gen, gen, gennki, ki, ki (*kyou wa gennki*: I’m fine today.)” and “so, so, sore, sore, rero, rero, ro, rou, dameyamonnettararonnrenn, nani (*sore wa dame*: It’s not good for me.),” and she was unable to control the speed of her conversation and spoke too fast to have good pronunciation. Stuttering was often observed in spontaneous speech and repetition, but was relatively lesser during the naming and reading of Kanji and Kana. In addition, she could not understand simple replies or responses from her conversation partners, and frequently asked for repetition by saying “What? What?” Because of such interruptions of conversations, she eventually fell silent, and could hardly have conversations with other people. From around this time, clock drawing and cube copying became difficult and were accompanied by constructional disabilities, and she frequently lost her way, even in familiar places.

At the age of 77, at around the same period during which she began speaking to mirrors, she developed the symptom of talking to people in magazines. Conversely, she continued to have communication difficulties in interpersonal conversations, because even if she had started talking to others due to disinhibition, she could not understand their words and finally fell silent. However, in conversations with the person in mirrors or magazines, despite frequent stuttering, and phonemic paraphasia, she could continue talking expressively and fluently as if talking with someone over the phone. In addition, she also showed gestures, such as pointing or peering at her “conversational partner” in mirrors or magazines, paused in conversations to wait for the “conversational partner” to reply, responded, laughed aloud, and ended the conversations with satisfaction after talking for about 15–30 min each time. At that time, face recognition of family members and medical staff was intact, and she could recognize the reflection of her husband and medical staff in mirrors. However, she lost her self-recognition and asked “Who are you? How are you?” of her own mirror image. Throughout the clinical course of the disease, she could handle herself independently, and her ADL, including self-care, were intact.

### Neuroradiological findings

**Fig. 2:**
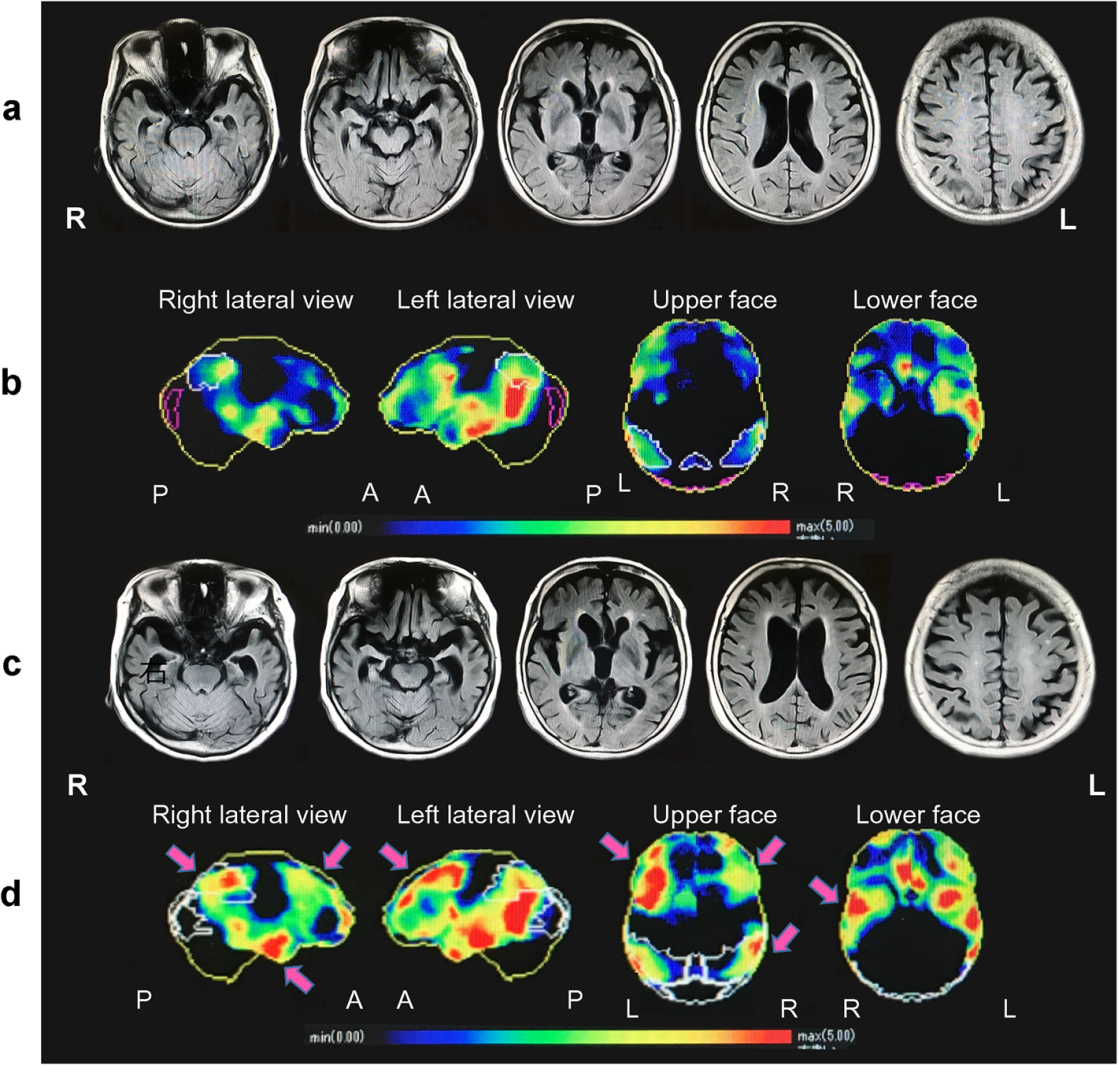
Brain MRI and cerebral blood flow findings at 75 and 77 years of age. At age 75, magnetic resonance imaging (MRI) showed cerebral atrophy of the temporal lobe, parietal lobe, and frontal lobe with dominance in the left cerebral hemisphere, and bilateral ventricular dilatation was also observed (**a**). In this image, the region and degree of decreased blood flow was objectively evaluated by displaying how many standard deviations the pixel value of the analyzed case deviates from the normal mean value using Z-score [Z-score = (normal mean value − case pixel value)/normal standard deviation]. The Z-score is indicated by 0 (blue) to 5 (red); the higher the value, the stronger the decrease in blood flow. Single-photon emission computed tomography (SPECT) with *N*-isopropyl-p-(iodine-123)-iodoamphetamine (IMP) revealed a reduction of cerebral blood flow in the left hemisphere with slight predominance in the frontal, temporal, and parietal lobes and posterior cingulate gyrus to cuneus (**b**). At age 77, the brain atrophy extended to the temporal-parietal lobe of the right hemisphere, with bilateral frontal lobe atrophy (**c**). Consistently, blood flow reduction was spread over a wide area in the right hemisphere, and a reduction in blood flow is observed in the bilateral frontal lobe, temporal lobe, and parietal lobe. Arrows demonstrate the areas of the region where hypoperfusion has expanded (**d**)

Figure 2 shows the images of the brain of the patient acquired using magnetic resonance imaging (MRI), as well as her regional cerebral blood flow, as assessed using single-photon emission computed tomography (SPECT), at the ages of 75 and 77. At the age of 75, the brain MRI showed atrophies in the superior temporal lobe, parietal lobe and frontal lobe of the left hemisphere, and enlargement of the left and right lateral ventricles. SPECT revealed decreased regional blood flow in areas corresponding to the atrophies, and from the left superior frontal lobe to the temporal-parietal lobe, as well as from the posterior cingulate gyrus to the precuneus. At the age of 77, in addition to the progression of the atrophies and decreased blood flow in those areas, the atrophies, and decreased blood flow in the frontal and temporal-parietal lobes of the right hemisphere had progressed rapidly. The patient and her family were informed of the test results, and she continued exercise and cognitive rehabilitation for maintaining her ADL.

## Discussion and conclusion

We experienced a rare case of an old woman who manifested Wernicke’s aphasia with stuttering during the course of dementia caused by Alzheimer’s disease; she also exhibited the mirror phenomenon, which appeared as an impairment of the visuospatial ability. Although she could hardly have conversations with other people because of severe aphasia, she could talk fluently with figures in mirrors or magazines. Aphasia with stuttering and mirror phenomenon, as in this case, rarely appears together during the course of neurodegenerative disease. In addition, this report is valuable because it presents a case in which the underlying mechanisms were examined using brain imaging and neuropsychological findings in a patient whose interpersonal conversations and conversations with mirror images exhibited very different features.

The patient’s relatively early symptoms of verbal disturbance were characterized by difficulties in single-word retrieval, repetition of sentences and phrases, and the presence of phonologic errors, which are consistent with the diagnostic criteria of the logopenic variant of progressive primary aphasia [6] and considered the Wernicke type of aphasia. Most logopenic variants are typically associated with the pathological finding of Alzheimer’s disease. In a study reporting autopsies of 58 patients with primary progressive aphasia, Alzheimer pathology accounted for 12% of the agrammatism type, 33% of the semantic type, and 56% of the logopenic type, which is the most common type of progressive aphasia caused by Alzheimer pathology [7]. The current patient had a disorder of the left temporal lobe and parietal lobe observed on neuroimaging, which was consistent with logopenic aphasia.

In contrast, very few cases of aphasia with AS in nonvascular dementia have been reported [8, 9], and none of their disease courses have been traced. The pathology of AS may involve the closed-loop motor system (cerebral cortex–thalamus–basal ganglia–cortex), and a study involving positron emission tomography (PET) based on the findings of the hypometabolic regions of patients with AS suggested the existence of a phonological loop in the anterior region of the left frontal lobe and temporal lobe [10]. However, as reported previously by us, Wernicke’s aphasia may be accompanied by AS because of injury of the region from the temporal-parietal lobe to the occipital lobe in the left hemisphere [11]. Moreover, the symptom of continuing to talk despite AS may be related to the decrease in auditory comprehension that is unique to Wernicke’s aphasia, as well as the decreased function of the posterior region of the temporal lobe. In fact, in our case, atrophies were observed in the frontal lobe, superior temporal lobe, and parietal lobe of the left hemisphere, and SPECT revealed a decrease in regional blood flow from the left frontal lobe to the temporal-parietal lobe, as well as from the posterior cingulate gyrus to the precuneus. Wernicke’s aphasia might have appeared in this patient because of a functional decline of the posterior region, including Wernicke’s area, of the left brain. In addition, the anterior region of the left brain possibly caused AS.

Neuropsychological literature on mirrored self-misidentification suggests the involvement of the right hemisphere or frontal regions; however, further anatomical specification has been hampered by severe dementia and global cortical impairment in these patients [12, 13], and the detailed mechanism of the mirror phenomenon is still unknown. According to the functional MRI study that examined the neural mechanisms underlying mirrored self-face recognition, this condition is considered to involve multiple processes that integrate two perceptual cues: one is a temporal contingency of the visual feedback on one’s action related to cuneus; the other is matching with self-face representation in long-term memory, which is related to the right temporal, parietal, and frontal lobes [14]. The mirror phenomenon is sometimes considered as prosopagnosia with related symptoms, such as Capgras syndrome which is the inability to recognize one’s own face reflected in a mirror [15, 16]. However, in this case, the patient did not misidentify others’ reflections in the mirror or people around her; therefore, prosopagnosia alone cannot explain the mirror phenomenon for this patient.

It has been reported that mirrored self-image, MMSE score and the identification of the self are highly correlated, and that the manifestation of the mirror phenomenon is related to the severity of dementia [17, 18]. In our case, the mirror phenomenon was observed as the dementia progressed, and the symptom of talking to people in magazines appeared at the same time as did the mirror phenomenon. In addition, from the time of appearance of these symptoms, constructional disabilities, and the symptom of getting lost in familiar places were observed. These findings mean that visual cognition and visual memory declined as dementia progressed. SPECT performed during the same period revealed that, in addition to decreased blood flow in the left hemisphere, the decreased blood flow in the right temporal-parietal lobe had clearly progressed. This is similar to the case with vascular dementia exhibiting mirror phenomenon [19] and to the case with visual constructional disabilities [12] reported previously; based on these findings, we concluded that the right hemisphere might have played an essential role in the discrimination between oneself and others. These changes in the neurological imaging findings before and after the appearance of the mirror phenomenon showed that decreased brain function in the temporal-parietal region of the left hemisphere spread to the temporal-parietal region of the right hemisphere as dementia progressed, and that such regional progression may have caused visuospatial cognitive impairment. Furthermore, in our case, the atrophies and decreased blood flow in both the left and right frontal lobes also progressed. The further addition of declining judgment, behavioral disinhibition, and decreased self-awareness stemming from the two frontal lobe regions, may have led to the confusion between the self and others, as well as between real people and people in mirrors and magazines. This misconception could not be corrected because of the disturbance of visual feedback and visual memory as well as the inhibition of the change in thought process, leading to the manifestation of the mirror phenomenon.

Even though the patient used to be sociable and talkative, Wernicke’s aphasia with a significant decline in auditory comprehension progressed and she lost the ability to communicate with others. However, visual cognitive impairment, impaired judgment in the discrimination between herself and others and disinhibition also occurred, leading her to converse freely and fluently again with people in mirrors and magazines; i.e., with unreal conversational partners, such as mirror images or people in magazines, she could enjoy engaging in smooth conversation. Thus, if the auditory comprehension is impaired, the phonemic stimuli from the outside may in turn hinder the communication of patients with aphasia. From this point, we present a very intriguing case that can be used for studying conversation in patients with severe aphasia.

In conclusion, this report is invaluable as it is a case in which the underlying mechanisms of mirror image and aphasia associated with dementia were continuously investigated using brain images and neuropsychological findings.

As limitations of this report, this case is clinically consistent with Alzheimer’s disease, although no definitive diagnosis of Alzheimer’s disease to date has been made. Even for primary progressive aphasia, which is relatively well-studied at the single patient level, no clinical pattern was pathognomonic of a specific neuropathology type, highlighting the critical role of biomarkers for diagnosing the underlying disease [7]. Therefore, for diagnosing dementia with rare symptoms, the ideal tool is biomarker evidence, such as cerebrospinal fluid amyloid β, T-tau and P-tau, or PET tracer (11) C-Pittsburgh Compound-B (PIB) or PET imaging with 18F-AV45 (florbetapir). Finally, this study is a case report, and it is necessary to examine in detail whether the same thing will occur in similar cases in the future.

## Data Availability

Data sharing is not applicable to this article as no datasets were generated or analyzed during the current study.

## Abbreviations

ADL: Activities of daily living
AS: Acquired stuttering
MRI: Magnetic resonance imaging
PET: Positron emission tomography
SPECT: Single-photon emission computed tomography
MMSE: Mini-mental state examination
RCPM: Raven's coloured progressive matrices
FAB: Frontal assessment battery
RBMT SS: Revermead behavioural memory test screening score
WFT-C: Word fluency test-category
WFT-L: Word fluency test-letter
NPI: Neuropsychiatric inventory
